# Significantly earlier ambulation and reduced risk of near-falls with continuous infusion nerve blocks: a retrospective pilot study of adductor canal block compared to femoral nerve block in total knee arthroplasty

**DOI:** 10.1186/s12891-022-05735-6

**Published:** 2022-08-12

**Authors:** Yutaka Fujita, Hisashi Mera, Tatsunori Watanabe, Kenta Furutani, Haruna O. Kondo, Takao Wakai, Hiroyuki Kawashima, Akira Ogose

**Affiliations:** 1 Department of Orthopedic Surgery, Uonuma Institute of Community Medicine, Niigata University Medical and Dental Hospital, Uonuma Kikan Hospital, 4132 Urasa, Minami-Uonuma, Niigata 949-7302 Japan; 2 Present Address: Department of Orthopedic Surgery, Nagaoka Chuo General Hospital, 2041, Kawasaki-machi, Nagaoka City, Niigata 940-8653 Japan; 3 Department of Anesthesiology, Uonuma Institute of Community Medicine, Niigata University Medical and Dental Hospital, Uonuma Kikan Hospital, 4132 Urasa, Minami-Uonuma, Niigata 949-7302 Japan; 4 Present Address: Department of Anesthesiology, Niigata University Graduate School of Medical and Dental Sciences, 754, Ichibancho, Asahimachidori, Chuo-ku, Niigata 951-8211 Japan; 5 Division of Rehabilitation, Uonuma Institute of Community Medicine, Niigata University Medical and Dental Hospital, Uonuma Kikan Hospital, 4132 Urasa, Minami-Uonuma, Niigata 949-7302 Japan; 6 Division of Orthopedic Surgery, Niigata University Graduate School of Medical and Dental Sciences, 754, Ichibancho, Asahimachidori, Chuo-ku, Niigata 951-8211 Japan

**Keywords:** Total knee arthroplasty, Continuous infusion nerve blocks, Risk of falls and near-falls, Early ambulation recovery

## Abstract

**Background:**

Near-falls should be detected to prevent falls related to the earlier ambulation after Total knee arthroplasty (TKA). The quadriceps weakness with femoral nerve block (FNB) has led to a focus on adductor canal block (ACB). We purposed to examine the risk of falls and the earlier ambulation in each continuous infusion nerve block.

**Methods:**

Continuous infusion nerve block (FNB or ACB) was performed until postoperative day (POD) 2 or 3. Pain levels and falls/near-falls with knee-buckling were monitored from POD 1 to POD 3. The score on the manual muscle test, MMT (0 to 5, 5 being normal), of the patients who could ambulate on POD 1, was investigated.

**Results:**

A total of 73 TKA cases, 36 FNB and 37 ACB, met the inclusion criteria. No falls were noted. But episodes of near-falls with knee-buckling were witnessed in 14 (39%) cases in the FNB group and in 4 (11%) in the ACB group (*p* = 0.0068). In the ACB group, 81.1% of patients could ambulate with parallel bars on POD 1, while only 44.4% of FNB patients could do so (*p* = 0.0019). The quadriceps MMT values in the ACB group was 2.82, significantly higher than 1.97 in the FNB group (*p* = 0.0035). There were no significant differences in pain as measured with a numerical rating scale (NRS) and rescue analgesia through POD 3.

**Conclusion:**

ACB was associated with significantly less knee-buckling and earlier ambulation post-TKA, with better quadriceps strength. Our study indicated the incidence of falls and near-falls with continuous infusion nerve blocks, and support the use of ACB to reduce the risk of falls after TKA. It is suggested that a certain number of the patients even with continuous ACB infusion should be considered with the effect of motor branch to prevent falls.

**Supplementary Information:**

The online version contains supplementary material available at 10.1186/s12891-022-05735-6.

## Background

Total knee arthroplasty (TKA) is performed to treat osteoarthritis (OA) and rheumatoid arthritis by relieving pain and restoring function. Patients with TKA indication are at risk for falls preoperatively [1–4], related to lower extremity muscle weakness, proprioception, and physical function, knee instability and fear of falling [3, 5, 6]. Though TKA reduce the risk of falls [4, 7], postoperative falls remain a major problem and the fall prevention program among these population has received still attention [1, 3, 4, 8] along with the prognostic models for inpatient functional recovery after TKA [9, 10].

Pain management is important for the functional recovery and reduction of the loss of muscle strength [8, 11] in the immediate postoperative period and for minimization of the risk of complications, such as deep vein thrombosis [12, 13]. However, on the contrary, the risk for falls generated by earlier ambulation with overestimation of pain management would be concerned [14, 15]. Risk factors for falls in the early postoperative period have been reported for male, obesity, small-sized or rural hospitals, and types of anesthetic techniques [8]. However, the level of their evidence is less than moderate [8], especially the evidence on the early ambulation with pain management and the risk of falls after TKA is insufficient.

Peripheral nerve blocks (PNB) are increasingly used in contemporary multimodality pain regimens aimed at reducing the potential side effects of other analgesics, such as opioids [16]. Complications of PNB include neurovascular injury, systemic toxicity, and muscle weakness [16]. Continuous PNB, in which an infusion catheter is inserted percutaneously around the peripheral nerve, can be expected to have a long-term therapeutic effect, reported lower incidence of severe complications such as local infection and safe with catheters kept in place up to 76 h [16–18].

Femoral nerve block (FNB) has been widely used due to its ease of application and its excellent pain relief after TKA [19–25], while concerned with weakens quadriceps strength [26, 27] due to blocking the proximal femoral nerve which contains both motor and sensory fibers [28]. As the alternative, adductor canal block (ACB) can provide equally effective pain relief preserving quadriceps strength [29–32], because the blocking the nerve distally to that of the FNB can anesthetize only the purely sensory saphenous nerve in the adductor canal [26, 27, 33]. Many studies have suggested that the differences between FNB and ACB, whether the motor fiber is blocked or not, affects the earlier recovery of ambulation and the risk of falls during rehabilitation after TKA [14, 30, 34–49]. However, it should be considered some cases with ACB are anesthetized actually by not only purely sensory saphenous nerve but also affecting motor branch due to the spread of injectate and the anatomy of the adductor canal [50–53].

Reports on the incidence of falls in FNB and ACB have been inconsistent, some due to differences in postoperative activity restriction [15, 27, 29, 39, 42, 54–56]. Some studies have investigated the risk of postoperative falling, knee buckling/near falls or imbalance [15, 27, 42], and their outcomes are referred for the fall prevention practices [15, 27, 42]. Thus far, the superiority of ACB with lower risk of fall for single injection nerve block has been reported with observation of knee buckling/near-falls or imbalance [15, 27], but not investigated enough for continuous infusion nerve blocks. For continuous infusion also, while a significance in fall risk due to muscle strength is speculated, it has been reported no significance with the Tinetti scale which estimate the fall risk [42, 57]. More studied with continuous infusion, in terms of the risk of falls and early ambulation, are needed.

Here, we aimed to evaluate the risk of falls, the time to commencement of ambulation, and muscle strength between the two continuous infusion nerve blocks. We analyzed TKA cases performed under general anesthesia at our institution as rural core hospital, of which half received FNB and half received ACB, with continuous infusion out to POD 3. We investigated the day of the commencement of ambulation, the incidence of falls and near-falls in the first three PODs, quadriceps muscle strength of the patients who had been able to commence ambulation with parallel bars on POD 1, and the effect of pain relief between ACB and FNB during that period.

## Patients and methods

After approval for this retrospective study was obtained from the Institutional Review Boards of the authors’ affiliated institutions. Our institute has been established since 2015, and is a core hospital covering the primary to advanced medical care in the Uonuma region, rural area in Japan [58, 59].

We collected clinical data of patients who had undergone primary unilateral TKA under general anesthesia with FNB (October 2016 to September 2017) and ACB (October 2017 to March 2019) as part of routine anesthesia protocols. We excluded patients undergoing revision TKA because the postoperative pain and recovery associated with surgical invasion are significantly different from those of primary TKA. Demographics are shown in Table 1. The protocol for TKA patients at our institution uses a linear ultrasound transducer placed parallel to the groin to guide advancement of the needle to the femoral nerve (short axis in-plane approach) on the ventral side of the iliopsoas muscle (FNB) or the perineurium of the saphenous nerve in the adductor canal (ACB) to inject an initial bolus of 50 mg of 0.25% levobupivacaine with 10 mL saline. ACB was performed at the proximal end of the adductor canal, which was identified using the high-frequency linear probe of a LOGIQ e Premium system (GE Healthcare Japan, Tokyo, Japan) based on reference to a previous publication [53]. A catheter is advanced 3 cm distally through the needle and verified the perineural placement of the tip of the catheter injecting small amount air, and the solution which 200 ml of 0.25% levobupivacaine diluted with 100 ml of saline is infused continuously 4 ml/h until POD 2 to 3. Patients allow to inject a bolus of 3 ml of the solution postoperatively through a catheter as patient control anesthesia (PCA), but unable to repeat subsequent 30 min. All blocks were performed by anesthesiologists trained in the regional blocks for both FNB and ACB.

**Table 1:** Demographic data for FNB and ACB cohorts

Demographics	FNB	ACB	*p*
Age, y	75.7 ± 6.2	75.0 ± 7.1	0.63
Male, n (%)	6 (17)	8 (22)	0.77
BMI, kg/m^2^	24.4 ± 3.7	26.0 ± 2.8	0.03
Dementia, n (%)	6 (17)	4 (11)	0.52
Cardiovascular disease, n (%)	12 (33)	16 (43)	0.47
Neurological disease, n (%)	7 (19)	6 (16)	0.768
Pulmonary disease, n (%)	4 (11)	9 (24)	0.221
Diabetes, n (%)	7 (19)	9 (24)	0.78
Preoperative knee			
JKOM^a^ I (pain)	63.1 ± 25.4	57.7 ± 29.3	0.45
JKOM^a^ II to V (function)	49.9 ± 19.2	46.7 ± 21.8	0.536
Surgical time, min	135 ± 16	145 ± 21	0.026
Blood loss, mL	271 ± 128	355 ± 212	0.045

^a^Japanese Knee Osteoarthritis Measure:

JKOM I evaluates the pain component, with patients indicating their pain level on a continuous scale from 0–100, where 0 indicates "no pain at all" and 100 indicates "the most severe pain ever experienced"

JKOM II to V are for knee function components. JKOM-II asks eight questions about knee pain and stiffness, JKOM-III asks ten questions about activities of daily living, JKOM-IV asks five questions about general activities that require leaving the house, and JKOM-V asks two questions about the patient’s perception of their overall health and the contribution made by knee symptoms. Patients rate each of the 25 questions on a five-point Likert scale, with 0 indicating “best” and 4 indicating “worst”. A total score of 0 indicates little loss of function, while a total score of 100 indicates severe loss of function

Values were generated with two-sample two-tailed Student’s *t*-test and Fisher’s exact test. A *p* value < 0.05 was considered significant

Patient charts were reviewed to determine demographics such as age, sex, body mass index (BMI), and comorbidities, including the incidence of dementia, cardiovascular diseases (congestive heart failure, ischemic heart disease including ischemic coronary artery disease with or without stenting, prior myocardial infarction, arrhythmias [including atrial fibrillation with pacemaker], and valvular disease), neurological disorders (prior cerebral infarction, cervical spinal myelopathy, and lumbar stenosis) without lower limb paralysis, pulmonary disorders (COPD, bronchial asthma, restrictive lung disease including interstitial pneumonia) without the need for home oxygen therapy, and diabetes mellitus (DM). Preoperative knee pain and function was assessed and scored using the Japanese Knee Osteoarthritis Measure (JKOM) clinical score, a patient-reported disease-specific assessment of knee OA which reflects the specificity of the Japanese cultural lifestyle [60] with reliability and validity comparable to the Medical Outcome Study 36-Item Short-Form Health Survey (SF-36) and the Western Ontario and McMaster Universities Arthritis Index (WOMAC) [60–62]. JKOM consists of 5 domains: JKOM-I for the evaluation of pain with a visual analogue scale (VAS: 0 to 100, 0 being normal). JKOM II to V are for knee function components: JKOM-II has eight questions on knee pain and stiffness, JKOM-III has ten questions on activities of daily living, JKOM-IV has five questions on general activities that require leaving the house, and JKOM-V has two questions on the patient’s perception of their overall health and the contribution made by knee symptoms [60]. The replies to JKOM II–V are scored on a five-point Likert scale, with 0 indicating “best” and 4 indicating “worst.” To assess the patients’ general condition on POD 1, we calculated total intraoperative blood loss, including the intra-articular suction drainage volume measured postoperatively from indwelling drains that were removed on POD 1. Most surgeries were conducted by a single arthroplasty surgeon and 2 surgeries by one senior resident under his supervision. All surgery in this study was performed using a pneumatic tourniquet during the procedure. A skin incision was made at the midline of the knee and a medial parapatellar approach was used to expose the joint [63] using a medial pivot prosthesis (Evolution® Medial-Pivot Knee System, Microport Orthopedics, Tokyo, Japan). Preoperative planning and intraoperative support were performed using JIGEN, a 3D pre-operative planning system for TKA with jig simulation (Lexi Co., Ltd. Tokyo, Japan) [64]. We reviewed the surgical time considering postoperative pain due to pneumatic tourniquet.

We reviewed physical therapist and nurse records to detect falls and near-falls with knee buckling (excluding falls from bed) during rehabilitation and hospitalization after TKA. Physical therapy (PT) commenced on the morning of POD 1, consisting of two 20-min sessions per day, with all patients encouraged to bear weight and walk without knee immobilizer as soon as tolerable. The criteria for starting parallel bar ambulation training were knee stability and adequate pain control, i.e. PT assessment of knee stability during weight bearing including quadriceps muscle strength in the manual muscle test (MMT) (score, 0 to 5, 5 being normal) [65], which could not be measured in all patients due to pain associated with knee motion [66, 67]. Patients unable to exert muscle strength or maintain a standing position due to pain [68] were not allowed to proceed to parallel bar ambulation training until they were able to stand. However, even though inadequate quadriceps muscle strength, those able to maintain a standing position with hamstrings muscle strength and/or bony retention instead [69–71] were allowed to proceed to parallel bar ambulation training. The day of commencement of ambulation was recorded for each patient (Table 2). We classified knee buckling as a near-fall, indicating insufficient muscle output leading to instability in standing and ambulation [15]. Falls and near-falls with knee-buckling from POD 1–POD 4 were documented by physical therapists or nurses. Unobserved ambulation was not allowed until patients had already been observed to walk with the parallel bars without problems. They were then allowed to use a walker or cane unobserved, usually on POD 4 or later. Along with assessment of mobility, we recorded the MMT scores of the quadriceps muscle in those patients who had been able to ambulate on POD 1.

**Table 2:** Comparison of days to first ambulation

	Initiation of ambulation: n (%)			
	POD 1	POD 2	POD 3	POD 4
FNB (*n* = 36)	16 (44.4)	15 (41.7)	3 (8.3)	2 (5.6)
ACB (*n* = 37)	30 (81.1)	5 (13.5)	1 (2.7)	1 (2.7)

(*p* = 0.00575, Fisher’s exact test)

The pain intensity of patients at rest was evaluated using a numerical rating scale (NRS-11). The nurses asked patients to rate their pain intensity three times daily. The mean values on POD 1, 2, and 3 were assessed. The times of rescue analgesia till POD3 was counted. For rescue analgesia against postoperative uncontrollable pain, we have prepared whether diclofenac sodium suppositories or the intramuscular pentazocine injection under the nursing control, depending on weight, organ function, and/or comorbidity etc. of patient.

Event frequencies of falls/near-falls with knee-buckling were analyzed using Fisher’s exact test. Time to ambulation with parallel bars was compared using the Mann–Whitney *U*-test and Fisher’s exact test. All continuous data, including age, BMI, Surgical time, blood loss, preoperative knee pain and function with the JKOM score, postoperative NRS, and MMT scores were compared using a two-sample, two-tailed Student’s *t*-test. Some demographic data such as sex, comorbidities, and cases per number of rescue analgesia were compared using Fisher’s exact test. A *p* value < 0.05 was considered statistically significant. A power analysis is shown in the Supplementary Materials. All statistical analyses were performed with a modified version of R commander designed for specific use in biostatistics (EZR, Saitama Medical Center, Jichi Medical University, Saitama, Japan) [72].

## Results

A total of 80 primary TKA cases were performed in our institution between October 2016 and March 2019. Three FNB cases were excluded due to the use of spinal anesthesia, and three other FNB cases were excluded because of unintentional removal of the infusion catheter within 24 h after TKA. One case with ACB was excluded because cerebral infarction occurred on POD 1. The remaining 73 TKA cases met the inclusion criteria, for a total of 36 FNB and 37 ACB cases. Table 1 shows patient demographics; the ACB patients had a significantly higher mean BMI (*p* = 0.03), longer surgical time (*p* = 0.026) as well as greater blood loss (355 ± 212 mL) than that the FNB patients (271 ± 128 mL), albeit of borderline significance (*p* = 0.045).

The records of the physical therapists and nurses showed no falls. Near-falls with knee-buckling occurred within PODs 1–4 (FNB: 14/36 [38.8%], ACB: 4/37 [10.8%], *p* = 0.0068).

Statistically significant differences were observed between the groups in terms of initiation of ambulation in the immediate postoperative period (Table 2), with significantly more ACB patients ambulating on POD 1, at 1.27 (95% CI: 1.19–1.36) days, compared with 1.75 (95% CI: 1.47–2.03) days in the FNB group (*p* = 0.0019). MMT values for the quadriceps in patients who were able to ambulate with parallel bars on POD 1 in 30 cases in the ACB group was 2.82 ± 0.88 (95% CI: 2.49–3.14), significantly higher than the 1.97 ± 0.87 (95% CI: 1.48–2.45) in 15 cases in the FNB group, except for one case in the FNB group who could not be evaluated due to pain (*p* = 0.0035).

There were no significant differences between the two groups in pain level on POD 1–3 (NRS of FNB and ACB; 3.8 and 3.6 [*p* = 0.6] on POD 1, 3.4 and 3.6 [*p* = 0.52] on POD 2, 3.2 and 3.0 [*p* = 0.64] on POD 3. Cases per number of rescue analgesia till POD3 were not significantly different between the two groups (Cases of FNB and ACB; 20 and 26 on 0 analgesia, 8 and 5 on 1 analgesia, 6 and 6 on 2 analgesia, 2 and 0 on 3 analgesia, [*p* = 0.357]).

## Discussion

No falls were noted during rehabilitation in either group. In contrast, the two groups differed significantly with regard to near-falls with knee-buckling (FNB, 14/37 [37.8%]; ACB, 4/36 [11.1%]; *p* = 0.0068). Ambulation with parallel bars began significantly earlier in the ACB group compared with the FNB group. The mean MMT scores of the quadriceps of the patients who were able to ambulate with parallel bars on POD 1 were 2.82 (95% CI: 2.49–3.14) in the ACB group, significantly higher than the 1.97 (95% CI: 1.48–2.45) in the FNB group. When evaluated by NRS and rescue analgesia, no statistically significant difference in pain relief between the two groups was seen.

The patients with TKA indication are thought to be at a risk for fall. In the previous study, 48% of patients with TKA indication experienced falls in one year prior to surgery, and 30% of the non-indication group experienced falls during the same period, suggesting a relationship of these population with the lower limb proprioception and deficits in knee extension strength [3]. TKA surgery is expected to improve the function of patients' balance to prevent falls. With regard to the incidence of falls, there are reports of a significant reduction from 15% preoperatively to approximately 6% at one year and 3% at two years postoperatively [4], as well as a long-term reduction in fall risk with a hazard ratio of 0.66 [95% CI, 0.57 to 0.76] for elderly patients undergoing TKA compared with those who did not [7]. However, even the patients who have been postoperative for long term is still suffered from the risk of falls. It has been reported that 24% of patients indicated for primary TKA have had the experience of fall preoperatively, moreover, 45% of those who have fallen, and 20% of those who have not had falls preoperatively have fallen within the first year after surgery [1].With regard to the perioperative period of TKA, the falls are serious issue to be aware of in terms of patient recovery and safety management during hospitalization [38, 73–75]. Preoperative fall risk assessment and preventive intervention programs for the patients with TKA indication have received much attention [1, 3, 4, 8]. Recently, it has been pointed out that postoperative functional recovery after TKA is also that pain management are prognostic factors for the postoperative course during hospitalization, as well as the patient factors [9, 10]. Better pain management could do earlier postoperative rehabilitation for appropriate muscle training to maintain muscle strength and function, which could also help the fall prevention in the early postoperative period [8, 11].

On the other hand, the early ambulation with overestimation of pain management should be careful [14, 15], since the standing position itself increase the risk for falls. On the incidence of falls with continuous nerve block infusion in the perioperative period of TKA, Feibel et al. and Pelt et al. reported rates of falls after TKA with FNB continuous infusion of 0.7% (8 in 1190 cases) [35] and 2.7% (19 in 707 cases) [39], respectively. Moreover, Pelt et al. reported that 27% of their fall cases were falls while ambulating with assistance [39]. Wasserstrtein also reported 2.7% of the fall incidence in the early postoperative period after TKA, despite with the fall prevention program, the incidence with most of the patients with FNB continuous infusion [38]. These two reports suggest the limitations in fall prevention program with FNB continuous infusion. Bolarinwa et al. reported a fall rate of 0.13% (1 of 791 cases) with ACB compared to 1.3% (11 in 834 cases) with FNB and recommended ACB as the preferred regional analgesia for the TKA procedure [56]. However, it is noteworthy that all falls, even still in the case with ACB, had occurred by POD 2, despite in the fall prevention awareness program [56]. In our smaller study, the fall rate was 0% in both groups, despite the older patient population than others, without the knee immobilizer, our strict criteria for initiation of parallel bar gait training could prevent falls. Thacher et al. also reported low fall rates and suggested the importance of rehabilitation in a highly or closely monitored environment [15].

Moreover, the detection of the cases with knee buckling/near falls and imbalance is important for fall prevention in practice. We defined knee-buckling as a near-fall since it can lead to falls. According to the population-based study in the community, the symptoms of knee-buckling and instability are common and associated with inferior physical function, fear of falling, and actual fall in symptomatic populations [5, 6]. In our study, despite a significantly higher BMI in the ACB group, the frequency of knee-buckling was 14 patients (37.8%) in the FNB group compared to 4 (11.1%) in the ACB group (*p* = 0.0068). This number appears higher than that in the study by Thacher et al., which reported 17/129 patients (13.2%) in the FNB group and 3/150 patients (2.0%) in the ACB group [15]. This difference might be due to the older patient age at the time of surgery in our cases, differences in anesthesia administration (continuous infusion following bolus in our study vs. bolus alone in the previous report [15]), and possible differences in rehabilitation protocols and monitoring period.

In terms of the age of the population, our study is 5 years older than the patient population in their study [15] and may be higher risk for falls due to lower physical functions [8, 38, 73]. Moreover, the difference between continuous catheter infusion and a single bolus administration may be significant [15]. With regard to rehabilitation protocols and monitoring periods, Thacher et al.’s study monitored until POD2 and reported that 26 h may be the window during which patients who receive a single administration of either FNB or ACB are most vulnerable [15]. However, they found one case of falls in the FNB group on POD3, suggesting that the cases with knee buckling should be care of falls even beyond POD2 when the patient would at home or unmonitored [15]. Considering with the continuous infusion, we monitored until POD4. In the Japanese health care system, almost all patients remain hospitalized beyond POD2, allowing for adequate close monitoring. This difference in background due to health care system may also have affected possibly the detection rate of knee-buckling. In our present study, all of the near-falls with knee-buckling were confirmed by physical therapists, who monitored patients closely during the rehabilitation course. All knee-buckling episodes occurred within PODs 1–3, which may outline the minimum period when this close monitoring should be performed. Elkassabany et al. reported stopping the continuous infusion on the morning of POD 1 before starting physical therapy, and they could not detect the relative risk of fall according to the Tinetti fall risk scale [42, 57]. They reported also MMT values of the quadriceps was lower in FNB than in ACB, speculating the higher fall risk due to muscle weakness in FNB [42]. Although they did not report the actual incidence of near-falls with knee-buckling, it would be interesting to investigate the correlation of the incidence of near falls with the fall risk scales such as Tinelli scale in the future. Hopefully, the fall risk scale could be alternative where adequate close monitoring is not available.

A number of studies have suggested that ACB is associated with more prompt and better advancement of ambulation ability than FNB [41, 43, 44, 55]. Shah et al. reported that the ambulation ability, the timed up-and-go (TUG), the ten-meter walk test, and the 30-s chair stand test [26] all had significantly better results with continuous ACB than continuous FNB anesthetic infusions (51.81 s vs. 180.06 s, 67.0 s vs. 273.70 s, 5.25 vs.1.52, *p* < 0.001, respectively) after removal of the catheter [44]. In our study, the quadriceps muscle strength of most patients did not recover sufficiently for them to perform unsupported tests, including the TUG, the ten-meter walk test, and the 30-s chair test, which Shah et al. did on POD 1 and 2.　Our results support the idea that these kinds of tests are difficult to perform in the early period after TKA surgery [76]. The reason for the higher risk of falls for male patient during hospitalization has cited the overestimation of their ability [8, 74, 75, 77]. The overestimating the pain relief with blocks and promoting excessive ambulation in the early postoperative period may increase the risk of falls [8, 14, 15]. Mudumbai et al. reported that the ACB group was longer walking distance than the FNB group, although patients were assisted by front-wheel walker.

In terms of quadriceps strength, although ACB is considered to be an almost pure sensory block, Jaeger et al., in a study of healthy volunteers, using a single bolus of 30 mL of 0.1% ropivacaine, reported that the mean reduction in quadriceps strength compared with 30 mL of saline in the opposite limb was 8% with ACB and 49% with FNB [26]. Kwofie et al., using single 15 mL bolus of 3% chloroprocaine, reported that isometric contraction of the quadriceps declined by 11% at 60 min after ACB block in one leg and 95% with FNB in the contralateral leg in healthy volunteers [27]. Results of our study also showed that continuous PNB could affect quadriceps muscle strength, even using ACB. Because ACB regardless single-shot or continuous infusion, does have a slight motor effect, medical staff still requires close attention during the early postoperative period with recognizing the effect [50–53].

In our retrospective study, we evaluated the intensity of postoperative pain in addition to the safety of ACB using NRS on POD 1, 2, and 3. Most studies have supported the idea that ACB and FNB are equally effective in terms of pain relief [15, 29, 41, 54, 55, 78, 79]. Although some studies suggested that the ACB group consumes more opioids than the FNB, the difference is ultimately interpreted as minuscule or non-inferior [15, 29]. Our study showed no statistically significant differences in NRS-11 scores and the times of rescue analgesia between the two groups, indicating that ACB provided effective pain relief equivalent to that of FNB comparably to previous reports.

With regard to risk factors for perioperative falls, small-sized or rural hospitals have been indicated as well as age, muscle weakness due to FNB, men, and obesity [8].　The reason for the higher risk of falls in small-sized or rural hospitals has been speculated thought to be attributed to fewer staff and facilities [8]. We have shown that adequate monitoring until POD4 is sufficient to prevent falls even in a rural hospital. It is obvious but realistic such as extending the length of hospital stay with the higher and closely monitoring as well as the delaying the start of ambulation in order to prevent falls in small-sized or rural hospitals, regardless of which PNB is used. Further investigation is needed to develop the prognostic model for functional recovery, including fall prevention, in the early postoperative period after TKA.

We demonstrated significant differences in mobility when walking with parallel bars and in the frequency of near-falls with knee-buckling between patients receiving continuous FNB vs. ACB, with earlier ambulation and fewer near-falls with knee-buckling in the ACB patients. Our study with close monitoring till POD4 after TKA indicated no falls and the knee-buckling with near-falls, 38% of FNB and 11% of ACB continuous infusion nerve block, respectively. The differences between the two groups may be due to the lower effect of ACB on quadriceps strength. However, in earlier ambulation after TKA, the cases even with continuous ACB infusion would need careful monitoring to prevent from falls with recognizing a certain number of cases with the effect of motor branch [50–53].

The limitation of this study at first are its retrospective design and small number of patients. Because this pilot study was conducted at a rural core hospital, sample size was not large enough, but the purpose of this study has been achieved. Second, all procedures were performed by not a single surgeon and anesthesiologist. However, most surgeries were performed by a single surgeon, and each block by anesthesiologists was performed with a unified technique. Last, ACB was performed at the proximal end of the adductor canal, the anatomy of which might have been close to the position suggested as the femoral triangle block [53], because of consideration with the skin incision and the position of the pneumatic tourniquet for TKA surgery.

## Conclusion

Our study demonstrated that continuous ACB provides better ambulation recovery with equivalent analgesia to continuous FNB for TKA patients. Our study shows the safety and efficacy of continuous ACB as well as the importance of close monitoring for the fall prevention.

## Supplementary Information

**Additional file 1.**

## Data Availability

The data that support the findings of this study are available from the corresponding author, H.M., upon reasonable request.

## Abbreviations

TKA: Total knee arthroplasty
OA: Osteoarthritis
PNB: Peripheral nerve block
FNB: Femoral nerve block
ACB: Adductor canal block
POD: Post operative day
MMT: Manual muscle test
PT: Physical therapy
BMI: Body mass index
COPD: Chronic obstructive pulmonary disease
DM: Diabetes mellitus
JKOM: Japanese knee osteoarthritis measure
VAS: Visual analogue scale
NRS: Numerical rating scale
CI: Confidence interval
TUG: Time-Up-and-Go
