# Supplementary material for: Significantly earlier ambulation and reduced risk of near-falls with continuous infusion nerve blocks: a retrospective pilot study of adductor canal block compared to femoral nerve block in total knee arthroplasty
Source: BMC Musculoskelet Disord. 2022 Aug 12;23:768. doi: 10.1186/s12891-022-05735-6 (PMC9373377; doi:10.1186/s12891-022-05735-6)
Supplement: Supplementary file 1 — Additional file 1. [file 12891_2022_5735_MOESM1_ESM.docx]

**Supplementary Material**

**Power analysis of sample size**

A power analysis by a two-tailed test with the alpha error set at 0.05 was performed to determine whether our sample size was large enough. Based on the number of cases and the proportion of those initiating ambulation on POD 1, 30/37 cases (81.1%) in the ACB group and 16/36 cases (44.4%) in the FNB group, statistical power was calculated to be 0.868, which satisfies the ≥ 0.8 level suggested by Cohen for our sample size [1]. Based on episodes of near-falls in the form of knee-buckling, 14/36 cases (38%) in the FNB group and 4/37 cases (11%) in the ACB group, the statistical power was calculated to be 0.678, < 0.8. Conversely, assuming that both groups were equal in number and that episodes of near-falls in the form of knee-buckling would occur in this ratio, 38% in the FNB group and 11% in the ACB group, the sample size for each group required to satisfy the two-tailed test with an alpha error of 0.05 and power of 0.8 was calculated to be at least 47 cases.

1. Cohen J: Statistical power analysis for the behavioral sciences New York. *NY: Academic* 1988:p.54.
